# Supplementary material for: Structural basis for allosteric modulation of M. tuberculosis proteasome core particle
Source: Nat Commun. 2025 Apr 1;16:3138. doi: 10.1038/s41467-025-58430-0 (PMC11962144; doi:10.1038/s41467-025-58430-0)
Supplement: Supplementary file 1 — Supplementary Information [file 41467_2025_58430_MOESM1_ESM.pdf]

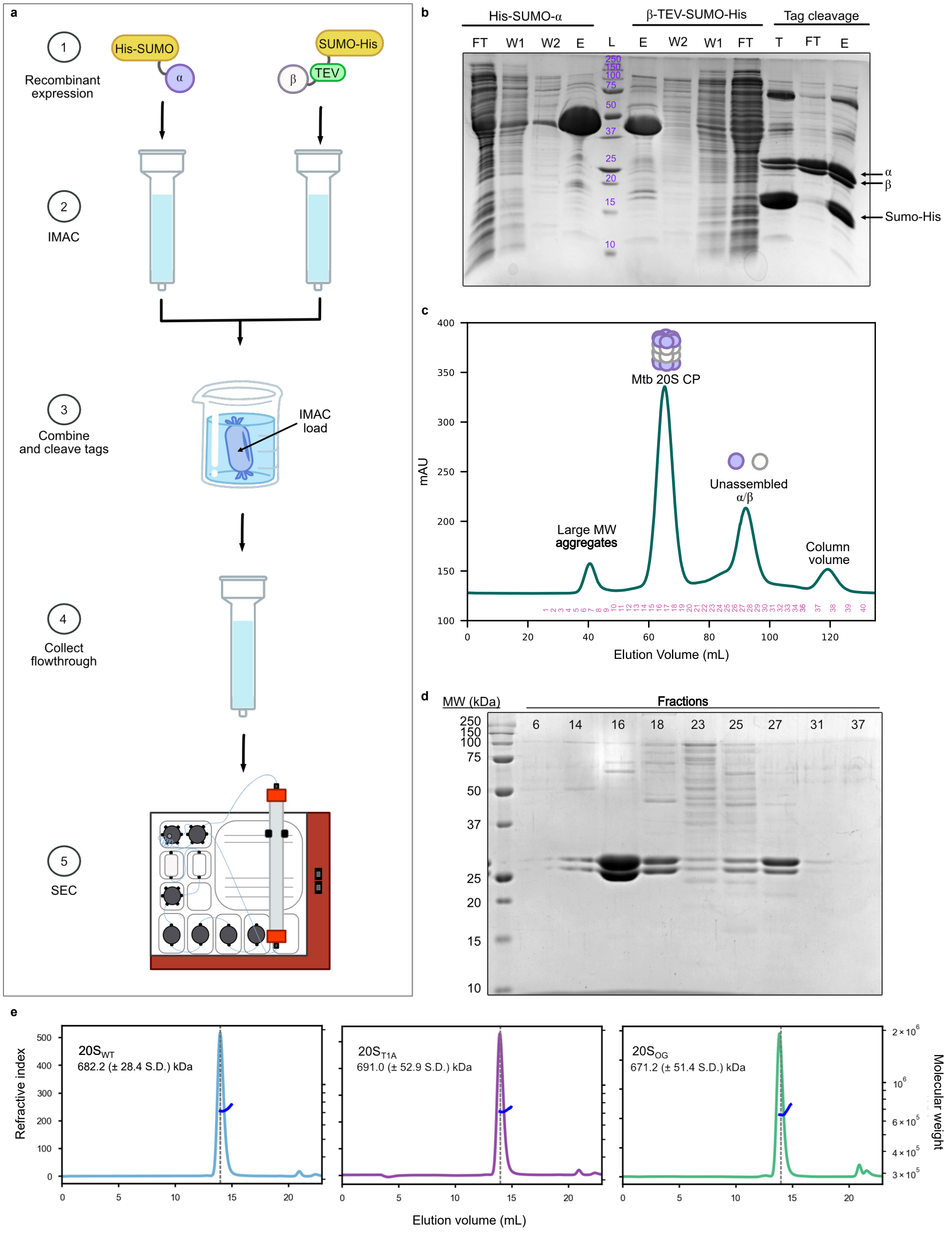

Supplementary Figure 1. Purification and oligomerization of the mature 20S CP. (**a**) The purification scheme for 20SP CP starts by isolating individually expressed α- and β-constructs using IMAC (**b**); before combining and purifying assembled core particle using SEC (**c**, **d**); and (**e**) SEC-MALS analysis confirmed the oligomerization state of each variant. Source data are provided as a Source Data file.

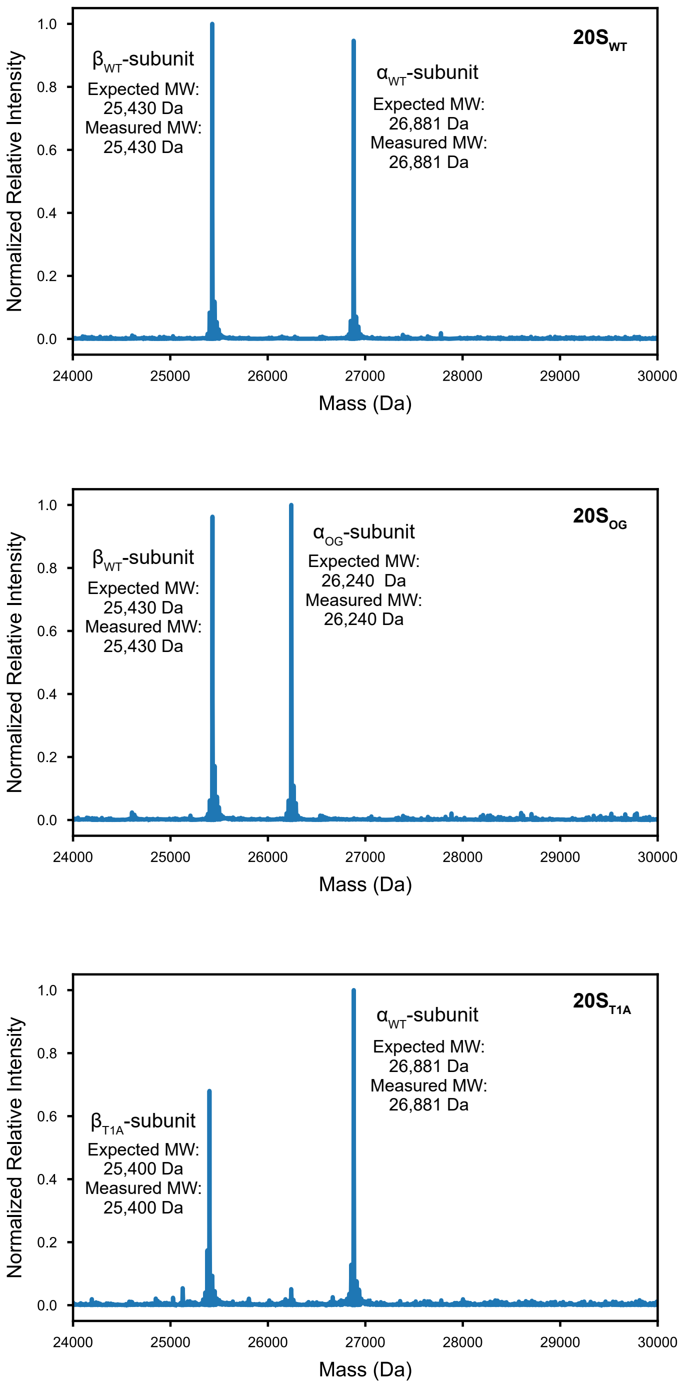

Supplementary Figure 2. Deconvoluted mass spectra of intact 20S CP variants reveal expected molecular weights for each subunit of interest (α_{WT}, α_{OG}, β_{WT} and β_{T1A}) within 1 Da. Spectra were summed over the two chromatographic peaks corresponding to the elution of each respective α- and β-subunit prior to deconvolution. Measured molecular weights and associated subunit are labeled.

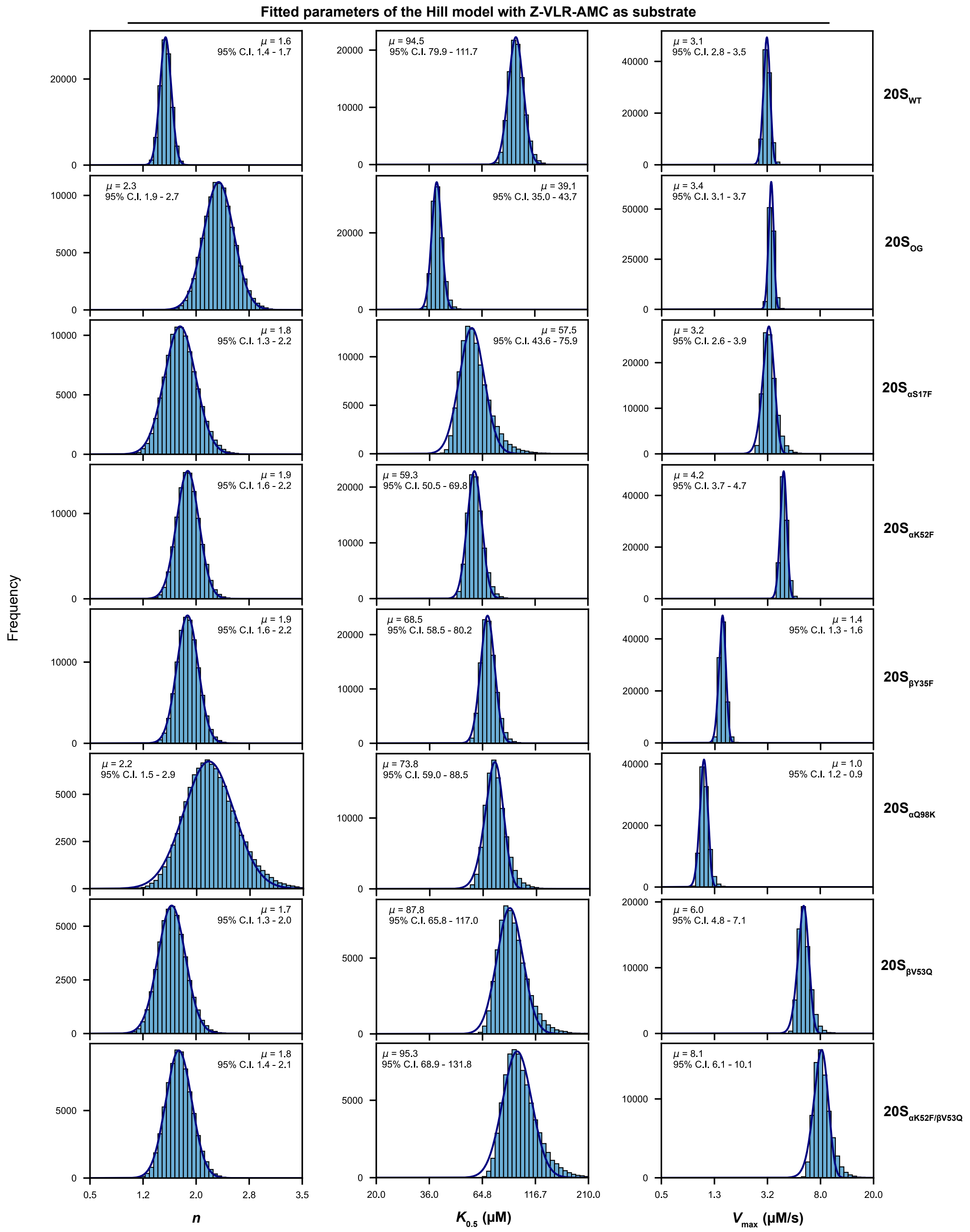

Supplementary Figure 3. Substrate hydrolysis by the 20S CP is well-described by the Hill model. Histograms representing 100,000 Monte Carlo simulations used to determine the fitted parameters and confidence intervals for *n*, $K_{0.5}$ and V_{max} values of the Hill model fit to the activity data generated using the peptide substrates, Z-VLR-AMC and LF2, for each 20S CP variant.

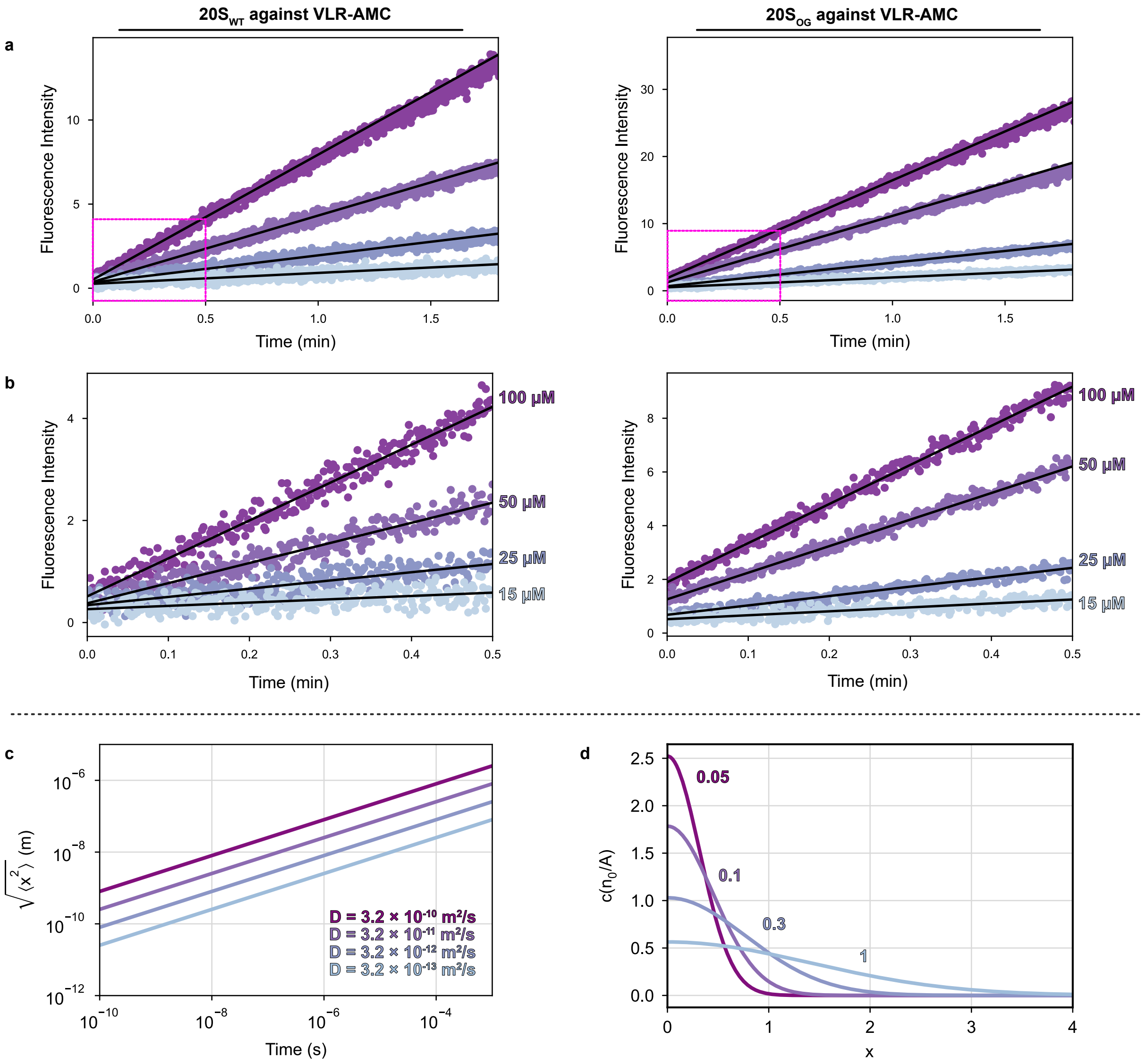

Supplementary Figure 4. Representative progression curves reveal that the gating residues do not inhibit Z-VLR-AMC degradation. (**a**) Reaction progression initially increases at a rapid rate and then decelerates for both $20S_{WT}$ and $20S_{OG}$ variants; (**b**) A 30-sec fitting window, indicated by the pink box in panel (a), was used to determine the initial velocities of substrate degradation. The calculated fits used to determine the rate of product formation are shown as black lines.; and (**c**) The root mean square distance $\sqrt{\langle x^2 \rangle}$ traveled by diffusing particles undergoing Brownian motion in time t, estimated using:

$$\sqrt{\langle x^2 \rangle} = \sqrt{2Dt}$$

which can be readily derived from the Einstein-Smoluchowski relation. We used a diffusion coefficient D of 3.2×10^{-10} m^2s^{-1}, estimated for Z-VLR-AMC using HydroPro[1] at room temperature, being reduced by 10-, 100-, and 1000-fold by the gating residues; and (**d**) The concentration profiles above a plane where a solute is diffusing. The curves represent the equation:

$$c(x,t) = \frac{n_0}{A(\pi Dt)^{1/2}} e^{-x^2/4Dt}$$

where each curve is labeled with different values of Dt. The units of Dt and x are arbitrary but chosen such that Dt/x^2 remains dimensionless. For instance, if x is measured in meters, Dt would be in square meters. On the microscopic length scale, for an ordinary diffusion coefficient $D = 10^9$ nm^2 s^{-1}, a Dt value of 1 nm^2 corresponds to $t = 1\times10^{-9}$ s. These plots demonstrate the effectiveness of diffusion over microscopic length scales. See ref[2]. Source data are provided as a Source Data file.

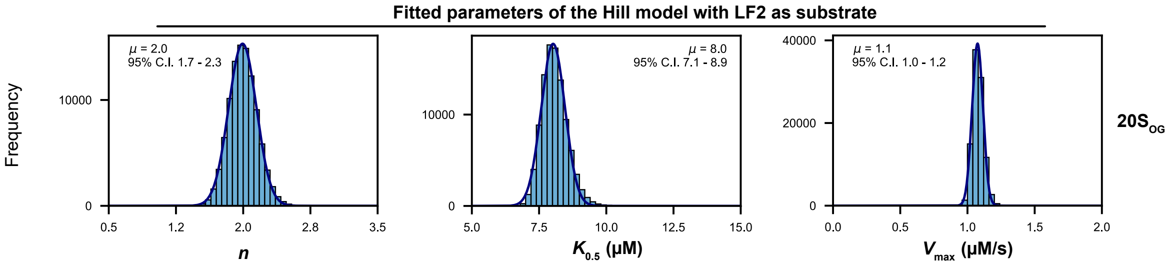

Supplementary Figure 5. Substrate hydrolysis by the $20S_{OG}$ is well-described by the Hill model. Histograms representing 100,000 Monte Carlo simulations used to determine the fitted parameters and confidence intervals for *n*, $K_{0.5}$ and V_{max} values of the Hill model fit to the activity data generated using the peptide substrates, LF2.

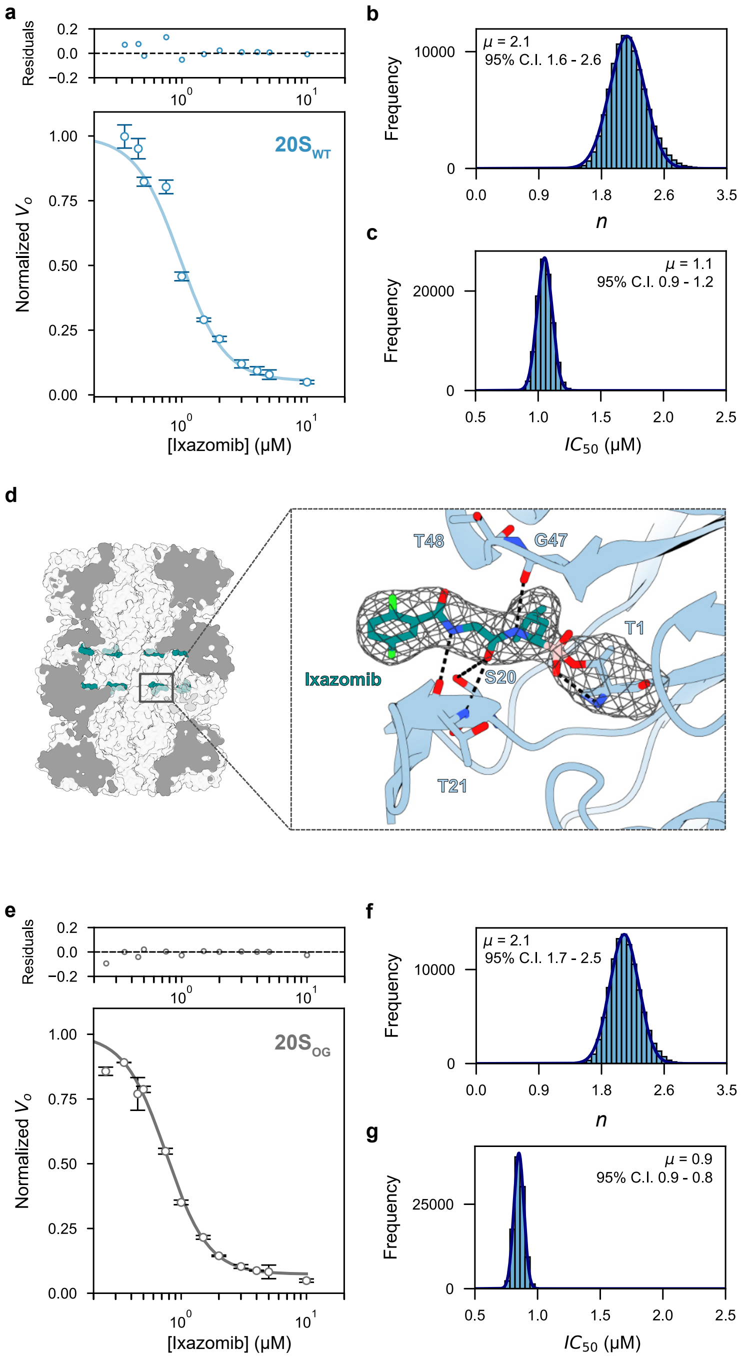

Supplementary Figure 6. The peptidyl boronate, Ixazomib, functions as a competitive inhibitor against the 20S CP. (**a**) Dose-response curve depicting the inhibitory effect of increasing ixazomib concentrations against the hydrolysis of the small tripeptide substrate, Z-VLR-AMC, by 20S$_{WT}$ fitted to the Hill model. The data points and error bars represent averages and standard deviation (95% C.I.), respectively, calculated on the basis of three technical replicates (n = 3); (**b**) Histogram representing 100,000

Monte Carlo simulations used to determine the Hill coefficient and respective confidence intervals; (**c**) Histogram representing 100,000 Monte Carlo simulations used to determine the IC_{50} value and respective confidence intervals; and (**d**) Cryo-EM structure of the $20S_{WT}$ CP bound to ixazomib (teal) with density of the small molecule represented as mesh. Density associated with ixazomib was noted in the active site of the CP and displays similar hydrogen bonding patterns to the previously solved structure of the human 20S CP bound to a related peptidyl boronate[3]. (**e**) Dose-response curve depicting the inhibitory effect of increasing ixazomib concentrations against the hydrolysis of the small tripeptide substrate, Z-VLR-AMC, by $20S_{OG}$ fitted to the Hill model. The data points and error bars represent averages and standard deviation (95% C.I.), respectively, calculated on the basis of three technical replicates (n = 3); (**f**) Histogram representing 100,000 Monte Carlo simulations used to determine the Hill coefficient and respective confidence intervals; and (**g**) Histogram representing 100,000 Monte Carlo simulations used to determine the IC_{50} value and the respective confidence intervals. Source data are provided as a Source Data file.

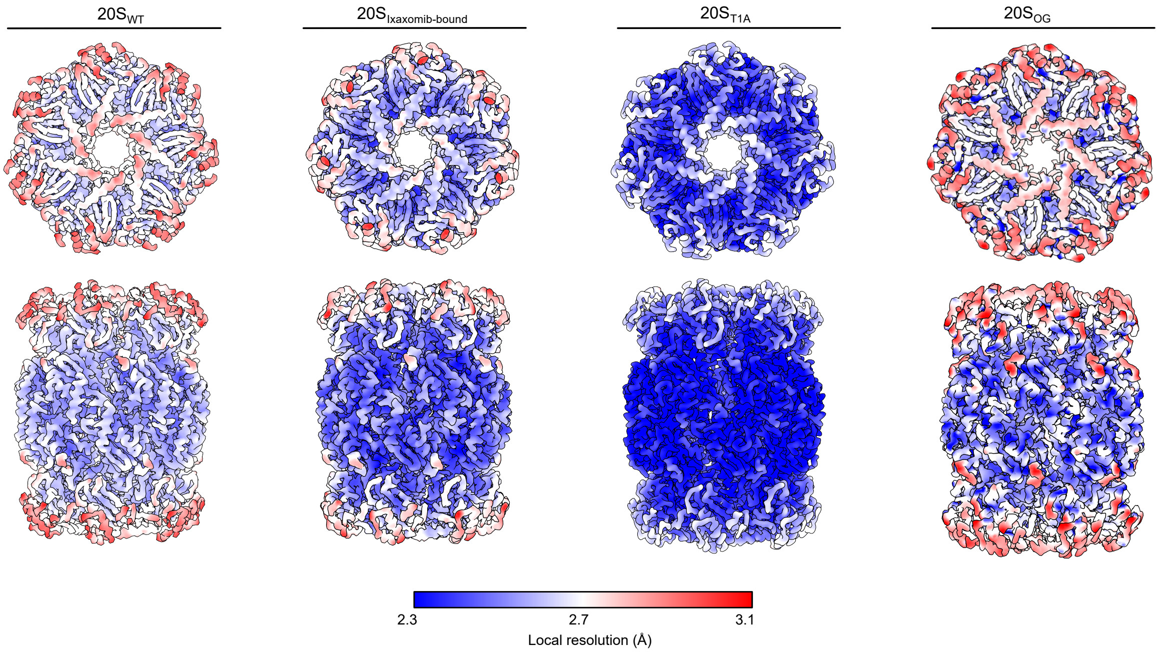

Supplementary Figure 7. Differences in local resolution across 20S CP variants suggest changes in solution dynamics. Cryo-EM reconstructed maps of each 20S CP variant coloured according to the local resolution defined by the colour bar. Top (above) and side view (bottom) of each particle map is shown.

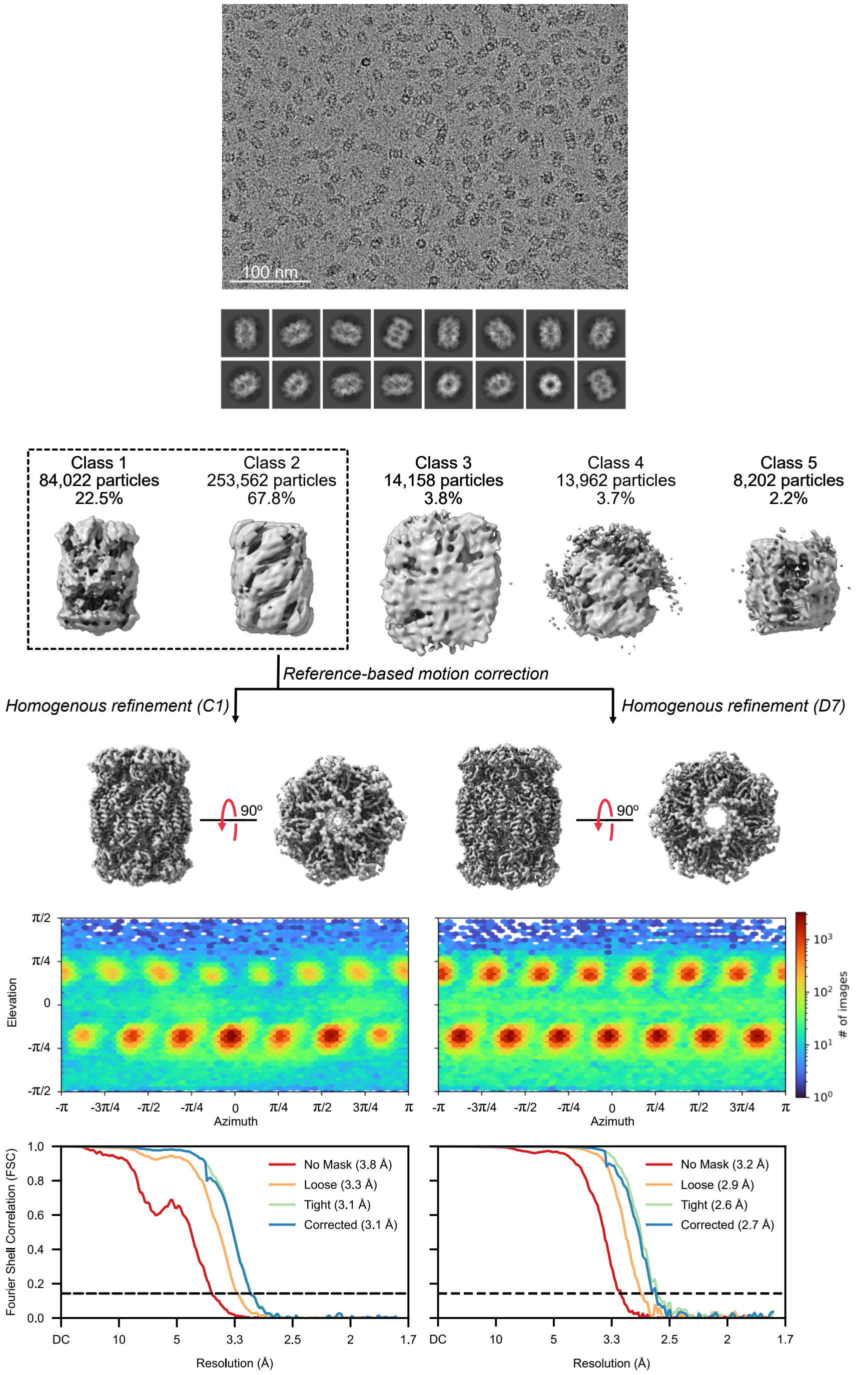

Supplementary Figure 8. Cryo-EM data processing pipeline for the wild-type 20S core particle. (a) Representative micrograph (b) Representative 2D classes (c) 3D classes after heterogenous refinement (d) Final consensus maps with C1 (left) and D7 (right) symmetry imposed (e) Viewing direction distribution plots for C1 (left) and D7 (right) refinements (f) Gold-standard Fourier shell correlation plots for C1 (left) and D7 (right) refinements. Source data are provided as a Source Data file.

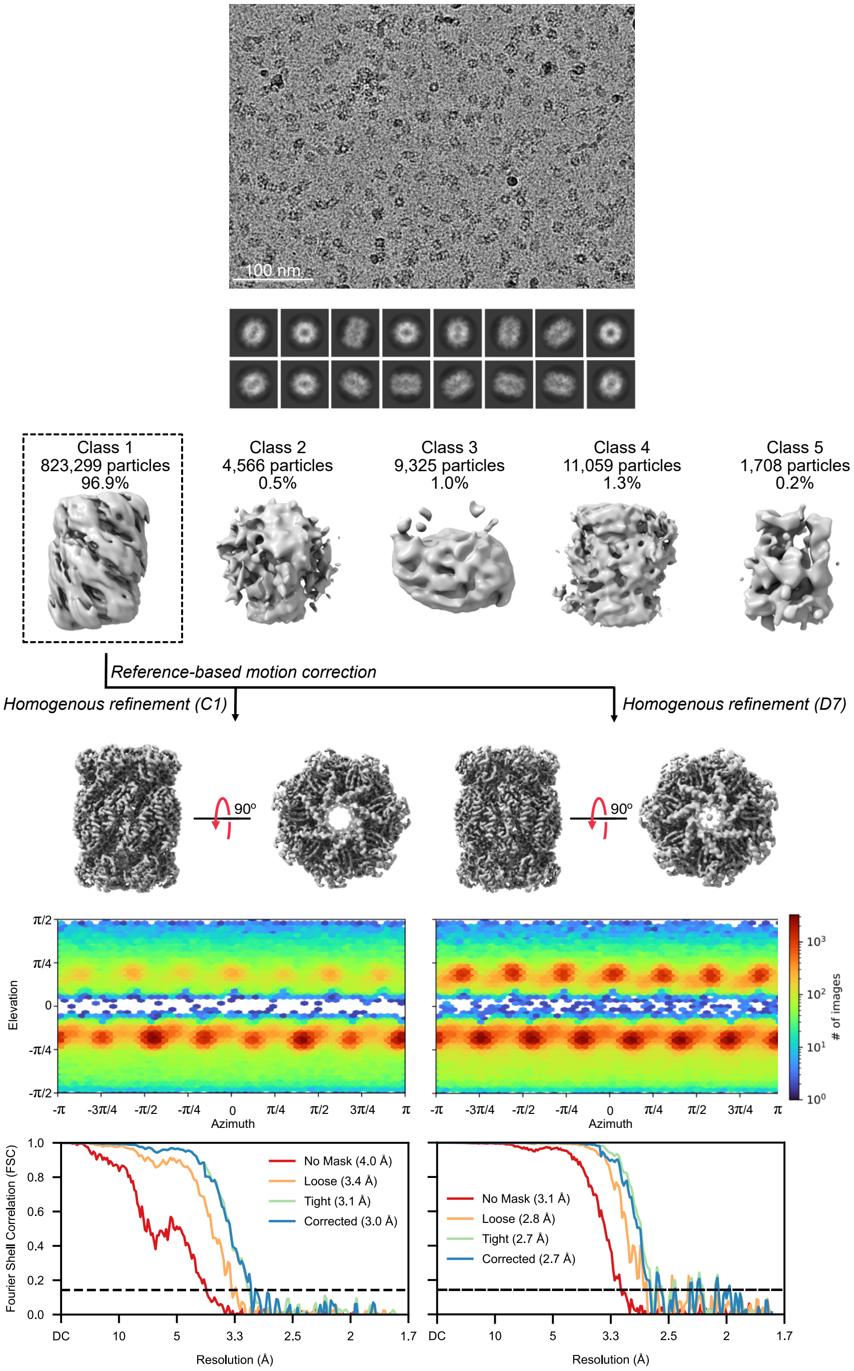

Supplementary Figure 9. Cryo-EM data processing pipeline for the open-gate 20S core particle. (a) Representative micrograph (b) Representative 2D classes (c) 3D classes after heterogenous refinement (d) Final consensus maps with C1 (left) and D7 (right) symmetry imposed (e) Viewing direction distribution plots for C1 (left) and D7 (right) refinements (f) Gold-standard Fourier shell correlation plots for C1 (left) and D7 (right) refinements. Source data are provided as a Source Data file.

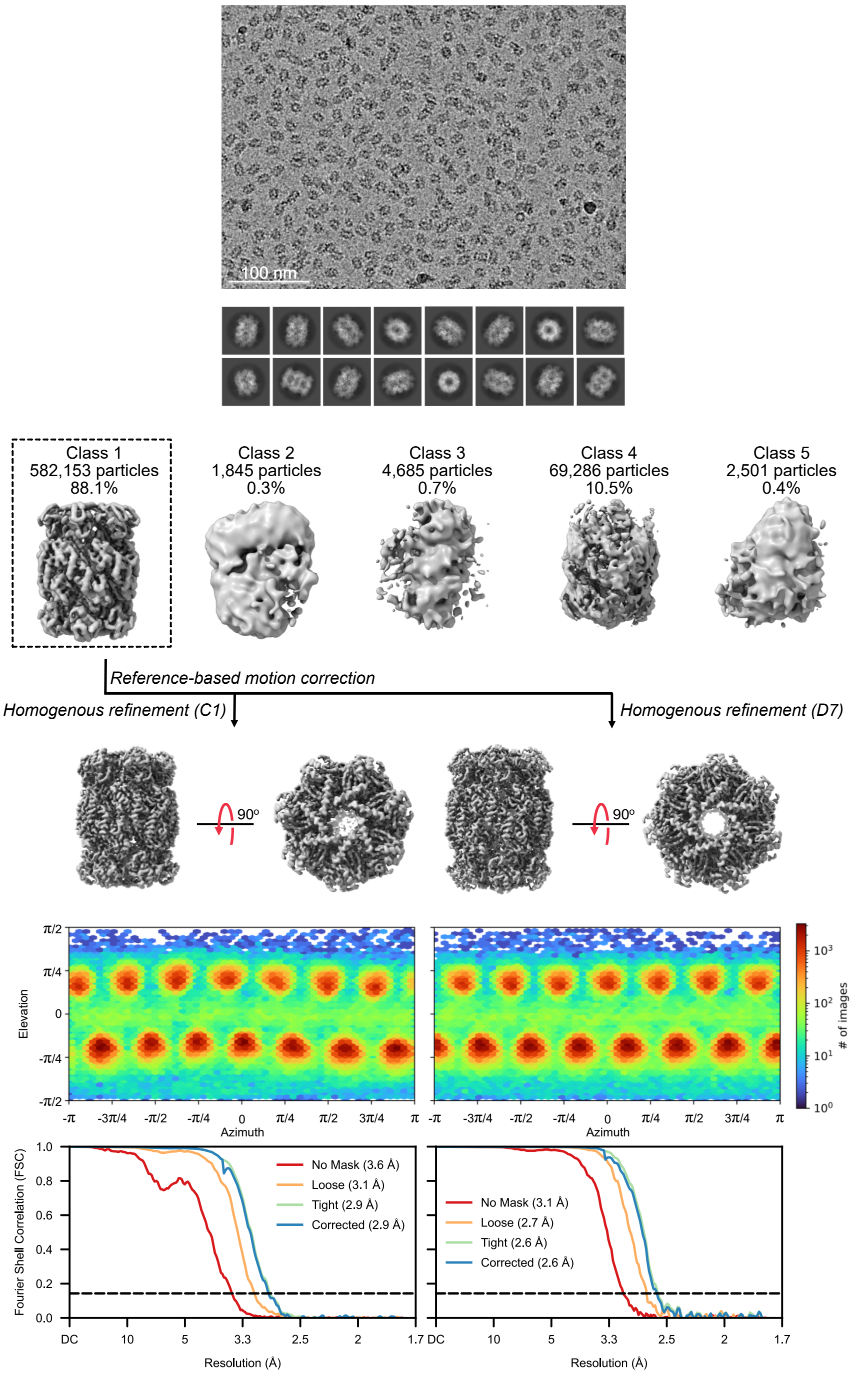

Supplementary Figure 10. Cryo-EM data processing pipeline for the T1A 20S core particle. (a) Representative micrograph (b) Representative 2D classes (c) 3D classes after heterogenous refinement (d) Final consensus maps with C1 (left) and D7 (right) symmetry imposed (e) Viewing direction distribution plots for C1 (left) and D7 (right) refinements (f) Gold-standard Fourier shell correlation plots for C1 (left) and D7 (right) refinements. Source data are provided as a Source Data file.

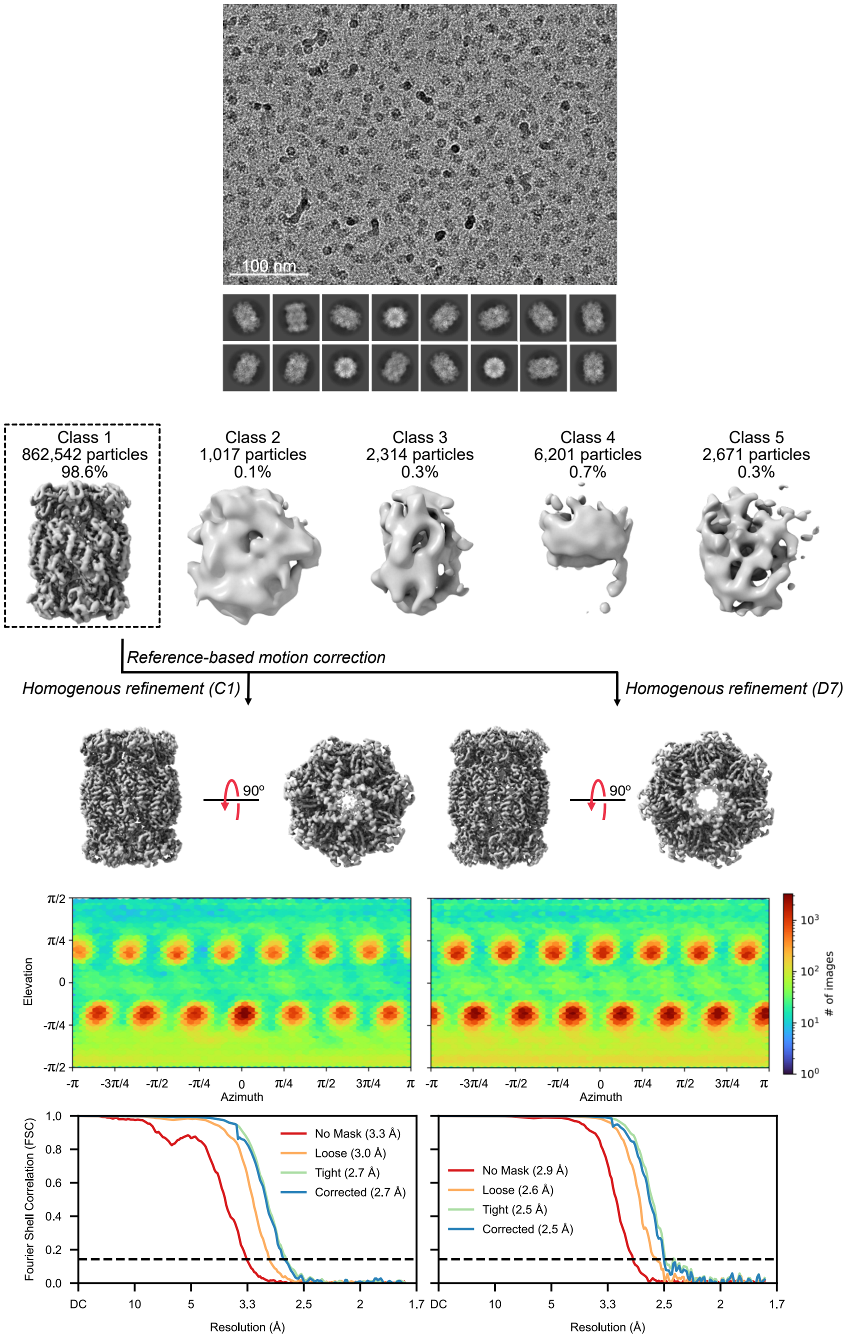

Supplementary Figure 11. Cryo-EM data processing pipeline for the Ixazomib-bound 20S core particle. (a) Representative micrograph (b) Representative 2D classes (c) 3D classes after heterogenous refinement (d) Final consensus maps with C1 (left) and D7 (right) symmetry imposed (e) Viewing direction distribution plots for C1 (left) and D7 (right) refinements (f) Gold-standard Fourier shell correlation plots for C1 (left) and D7 (right) refinements. Source data are provided as a Source Data file.

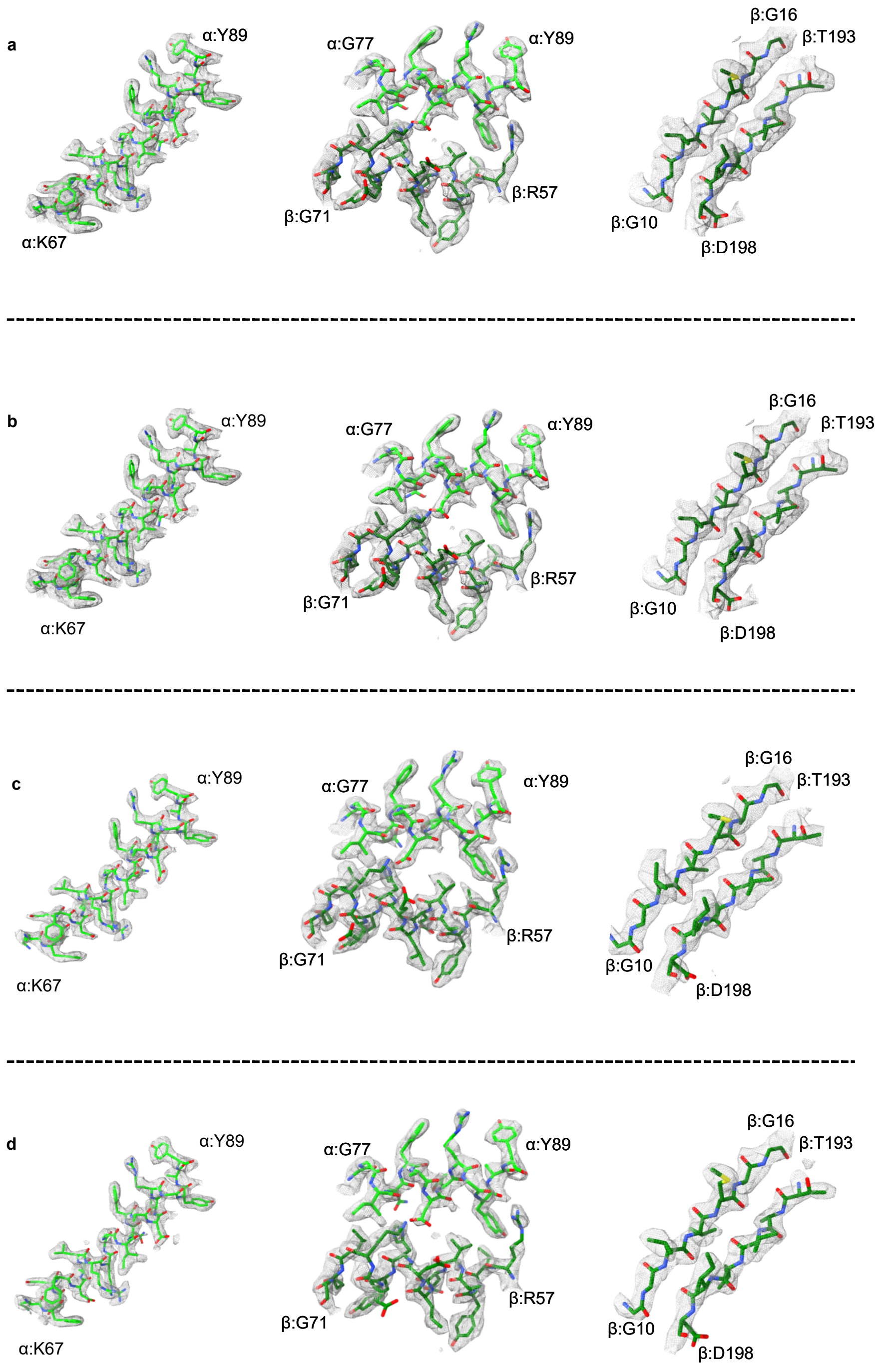

Supplementary Figure 12. Refined model to consensus map fit for selected regions (**a**) wild-type 20S core particle (**b**) the open-gate 20S core particle (**c**) T1A 20S core particle (**d**) Ixazomib-bound 20S core particle.

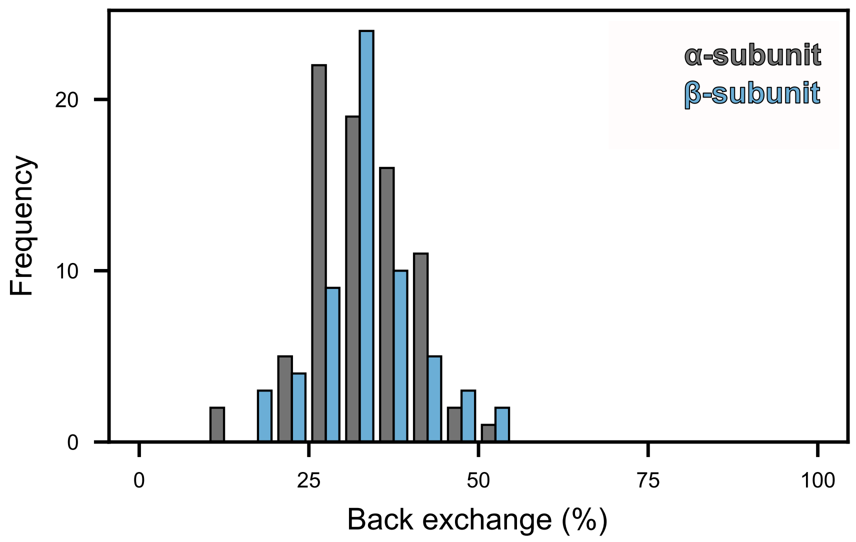

Supplementary Figure 13. Levels of HDX back-exchange across α- and β-subunit peptides. Histogram representing the distribution of α- and β-peptide back-exchange (H to D), expressed as the percentage of deuterium lost from the total number of deuterium incorporated as measured through maximally deuterated samples. Source data are provided as a Source Data file.

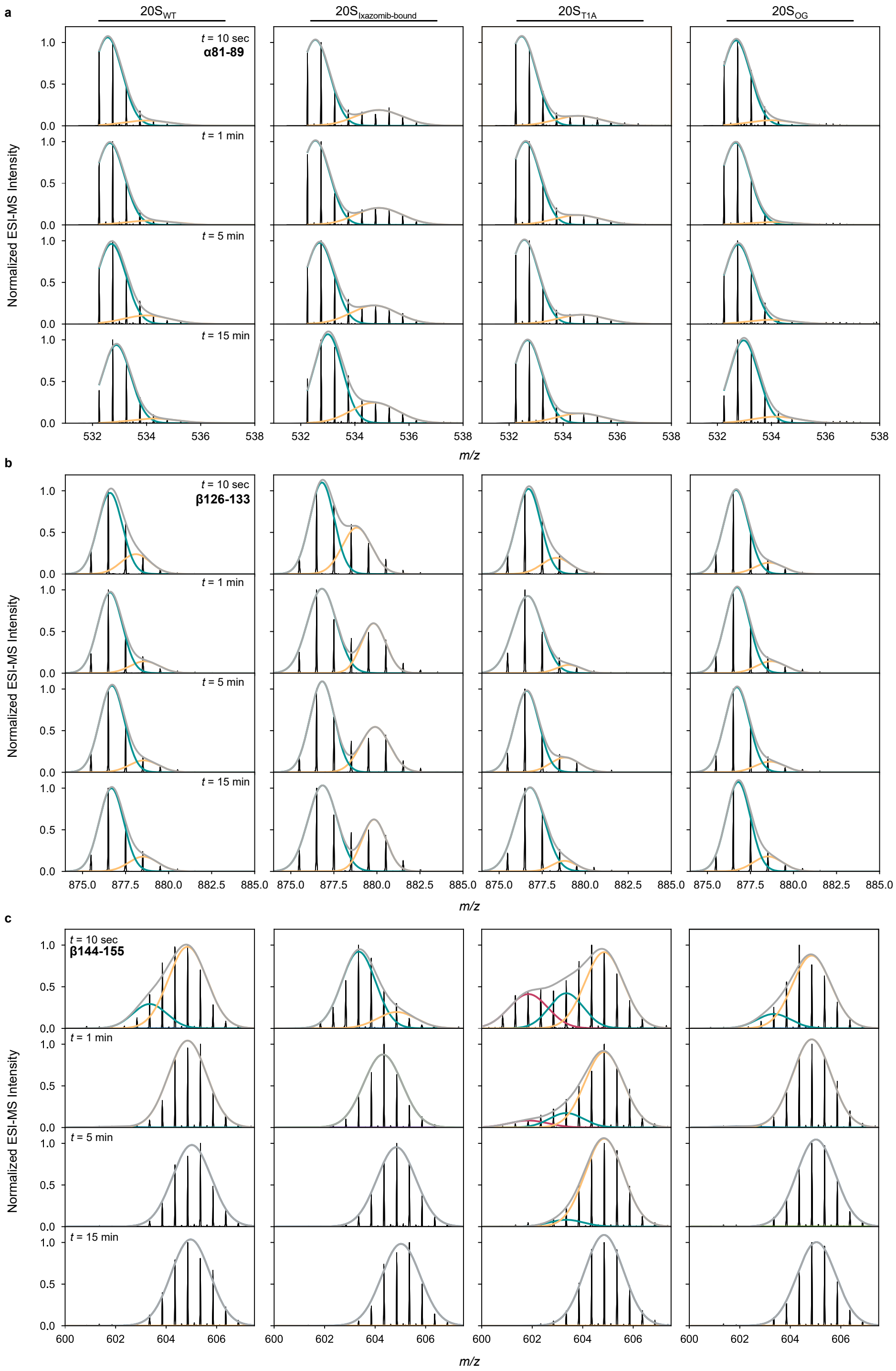

Supplementary Figure 14. HDX-MS isotopic envelopes of $20S_{\beta T1A}$ reveal an additional conformation compared to the other variants. HDX mass spectra of peptides from the switch helix I in unbound and ixazomib-bound $20S_{WT}$, $20S_{\beta T1A}$, and $20S_{OG}$ after 10 sec, 1 min, 5 min, and 15 min of D_2O exposure. The grey trace represents the sum of the Gaussian fits.

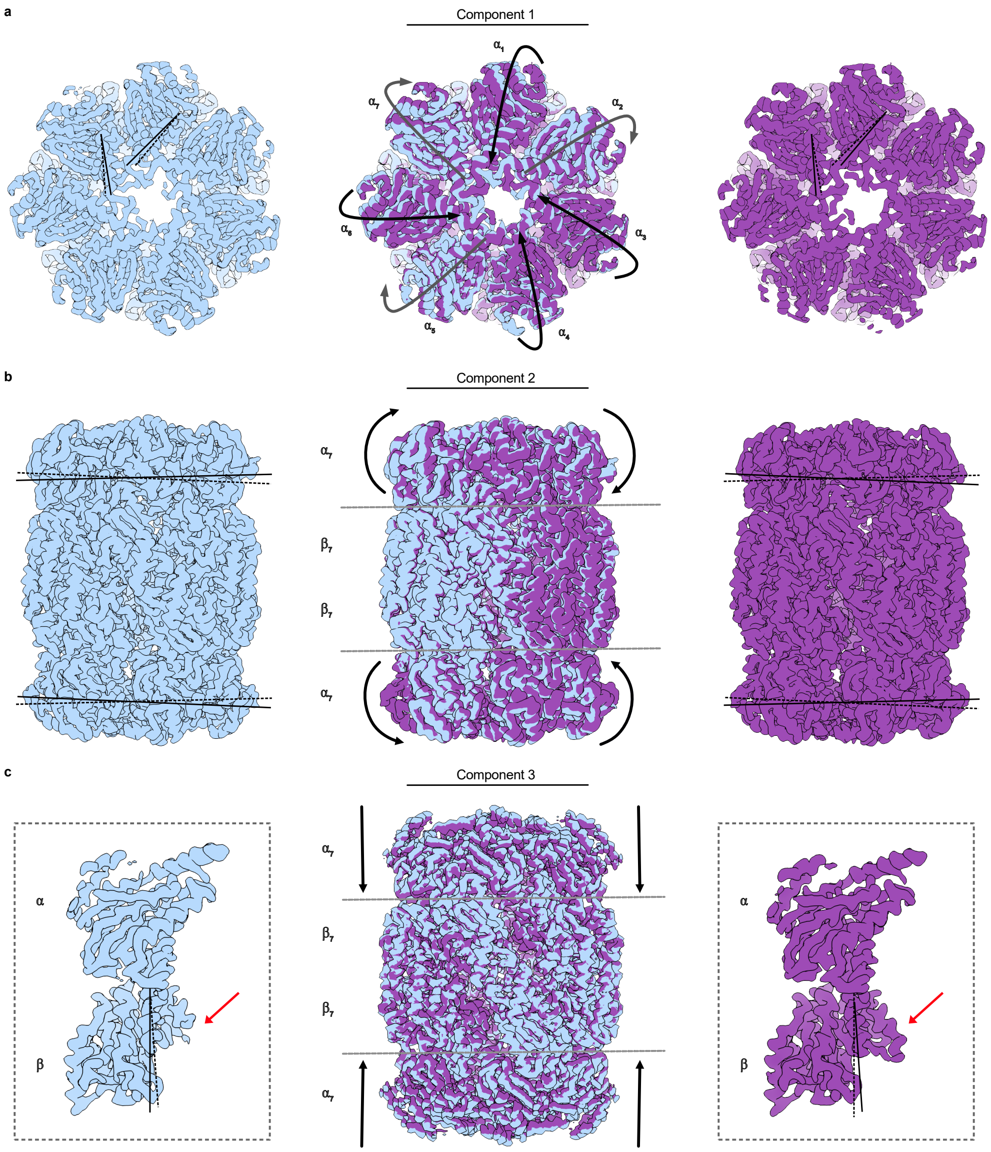

Supplementary Figure 15. 3D variability analysis of the D7 symmetry expanded particle of the 20S$_{\beta T1A}$ variant revealed three variability components of the 20S CP. Maps generated at negative (initial frame, coloured blue) and positive (final frame, coloured purple) latent coordinates along the variability components are overlaid for each component. Solid and dashed lines show the degree of motion between negative and positive frames. Arrows represent the direction of motion. The motions are also shown in Supplementary Movies 1-3 (**a**) The α-subunits compete for occupation of the central pore. (**b**) The α-rings rotate atop the β-rings. (**c**) Compression of the α-rings towards the barrel results in a shift in Switch Helix I and increased density of the Switch Helix II of the β-subunits, highlighted with red arrows, in the 20S$_{\beta T1A}$ structure.

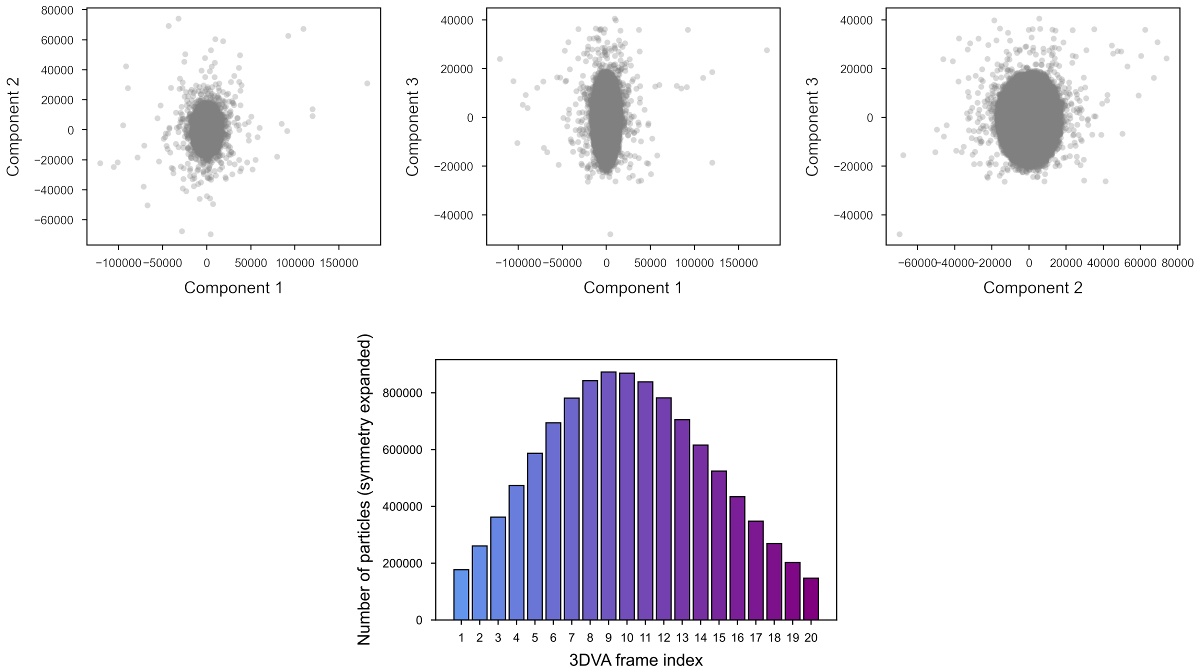

Supplementary Figure 16. Details on the 3DVA of the 20S$_{\beta T1A}$ variant. (Top) Distribution of particles along selected principal components from the 3DVA of 20S$_{\beta T1A}$ variant. The three plots show different combinations of the selected principal components; (Bottom) Number of particles in each of the 20 frames along component 3 of the 20S$_{\beta T1A}$ dataset. The particles were sorted using 3DVA with window=0, that is, discrete particle sorting with no overlap. Source data are provided as a Source Data file.

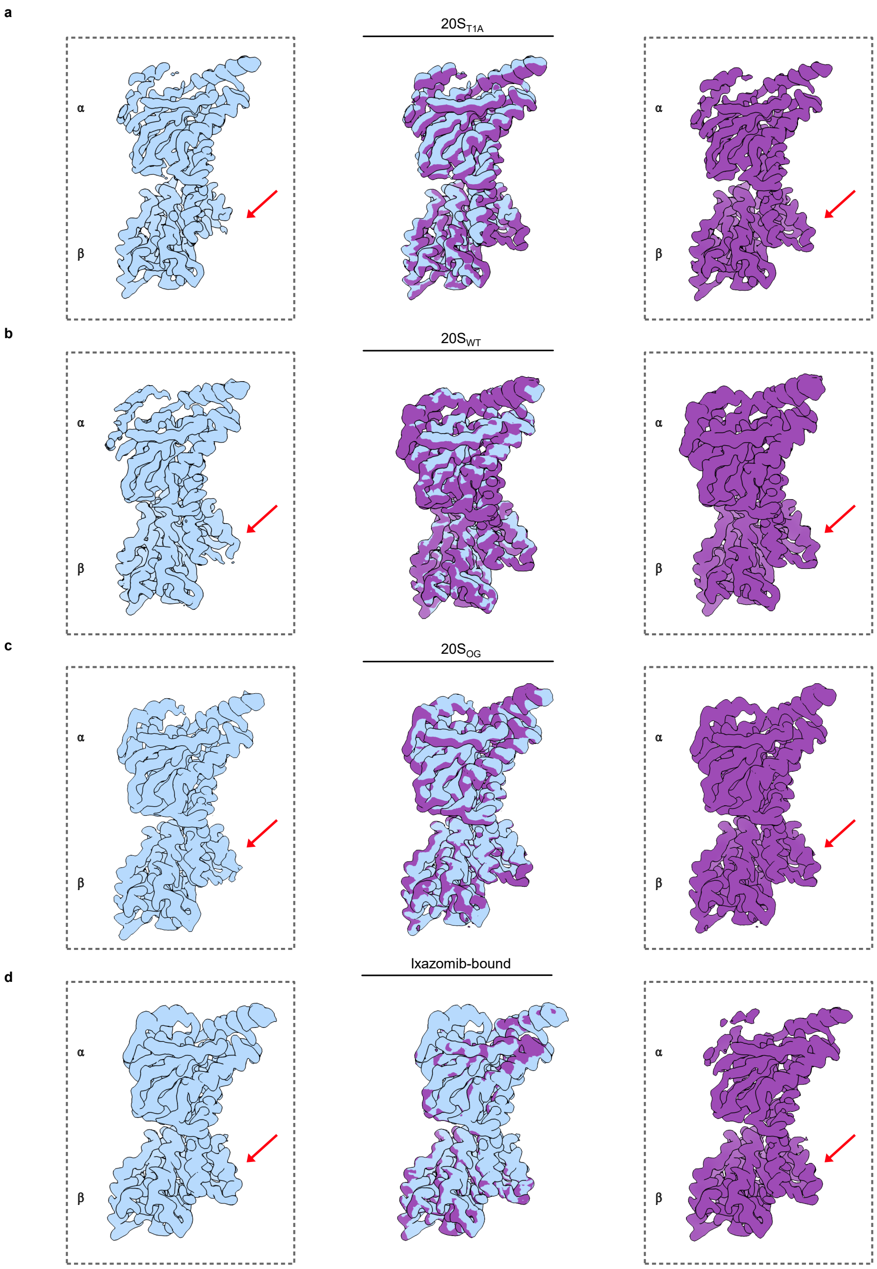

Supplementary Figure 17. 3D variability analysis revealed density changes in Switch Helix II resembling those seen in (a) the $20S_{\beta T1A}$, (b) $20S_{WT}$, and (c) $20S_{OG}$ structures, but not in (d) the $20S_{Ixazomib\text{-}bound}$ variant. Maps generated at negative (initial frame, coloured blue) and positive (final frame, coloured purple) latent coordinates along the variability components are overlaid. Switch Helix II of the β-subunit is highlighted with red arrows. See Supplementary Movies 3-6 for viewing the full motion.

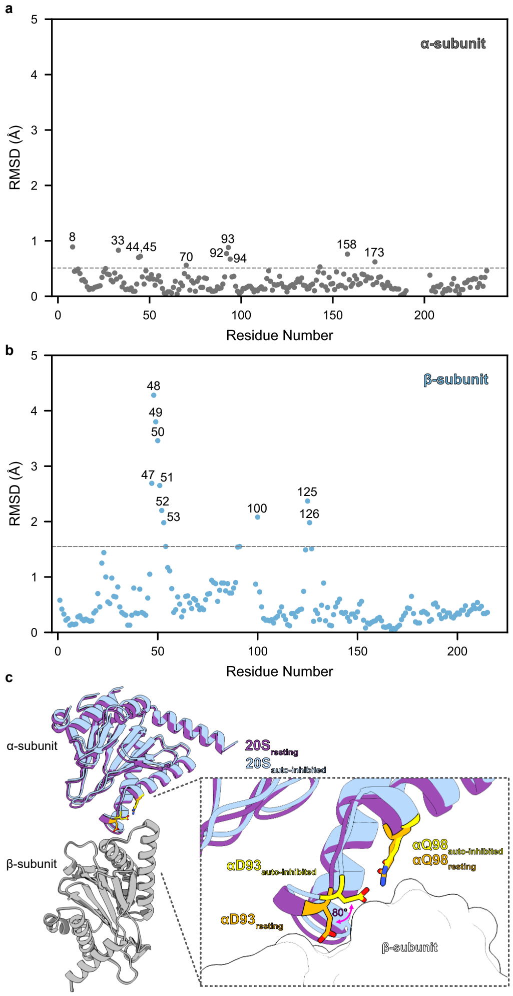

Supplementary Figure 18. Alignment of the $20S_{resting}$ and $20S_{auto\text{-}inhibited}$ states reveals the residues that undergo the greatest structural shifts between the two conformations. Cα RMSD scores showing the local structural differences between $20S_{resting}$ and $20S_{auto\text{-}inhibited}$ states in the (**a**) α-subunit or (**b**) β-subunit. The top 5% of residues showing the largest RMSD values are labeled with the respective residue number. A dashed line indicates the cutoff value separating the top 5% from the remaining datapoints; and (**c**) Between the $20S_{resting}$ and $20S_{auto\text{-}inhibited}$ states, αD93 undergoes a ~80° rotation. This residue is a likely point of allosteric communication across the α/β-interface.

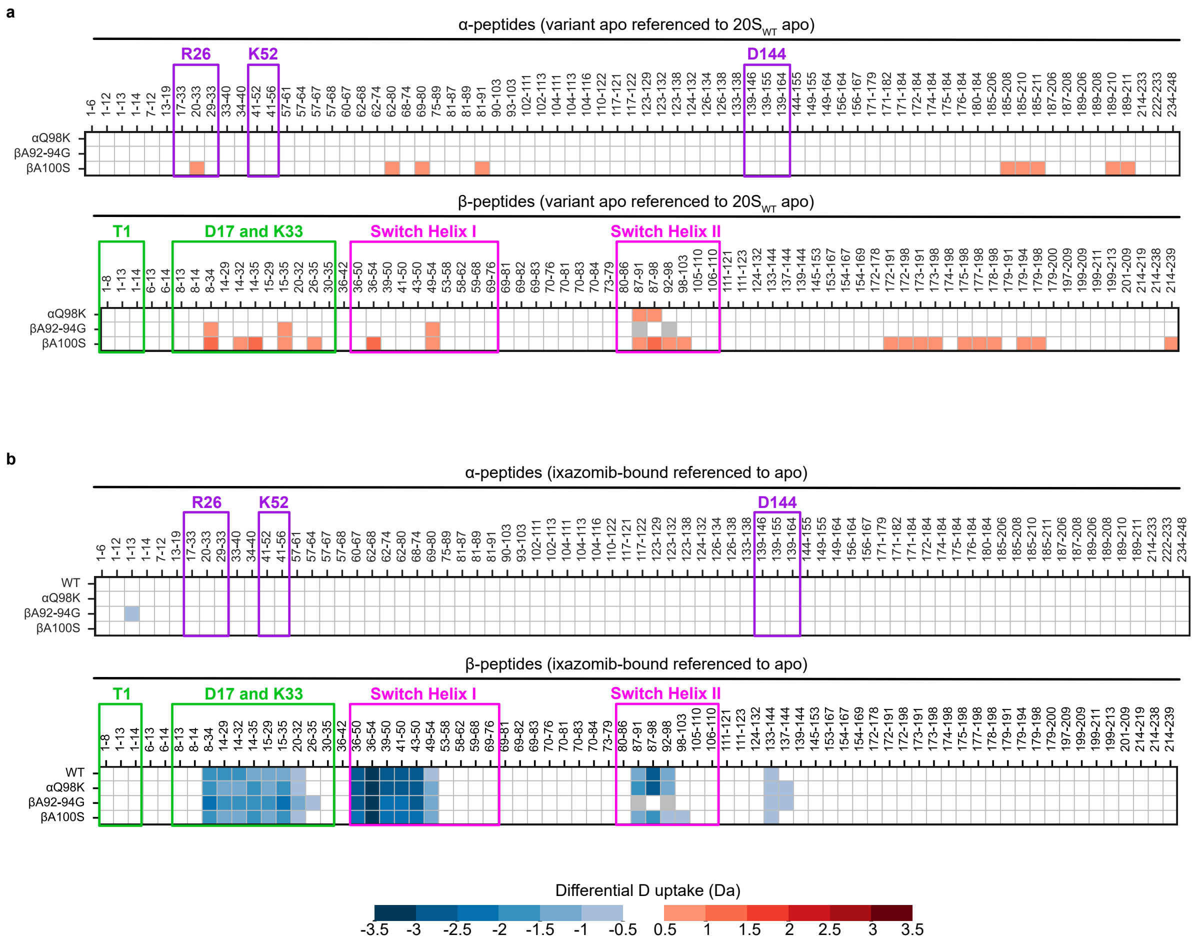

Supplementary Figure 19. The 20S variants designed to stabilize the 20S$_{\text{auto-inhibited}}$ state retain the capacity to bind substrate. Pulsed HDX-MS experiments were used to explore the conformational dynamics of the less active 20S$_{\alpha Q98K}$, and inactive 20S$_{\beta A93\text{—}94G}$ and 20S$_{\beta A100S}$ variants in the apo and ixazomib-bound states. Heat maps of relative deuterium uptake compared against 20S$_{WT}$ were generated, calculated on the basis of three technical replicates, where only changes larger than 0.5 Da were considered significant and were coloured according to the colour bar (bottom). (**a**) Each variant shows increased conformational dynamics in one or both Switch Helices compared to the 20S$_{WT}$ in the apo state. (**b**) The variants show similar D-uptake patterns to 20S$_{WT}$ in the ixazomib-bound state, except for a single peptide in Switch Helix II of the 20S$_{\beta A100S}$ variant, signifying that they can still bind substrate. The peptides generated for each subunit are listed across the top of the heat map and the associated variants are listed along the side. Gray squares indicate absent data. Peptides associated with the RP-binding sites of the α-subunit and switch helices of the β-subunit are labeled.

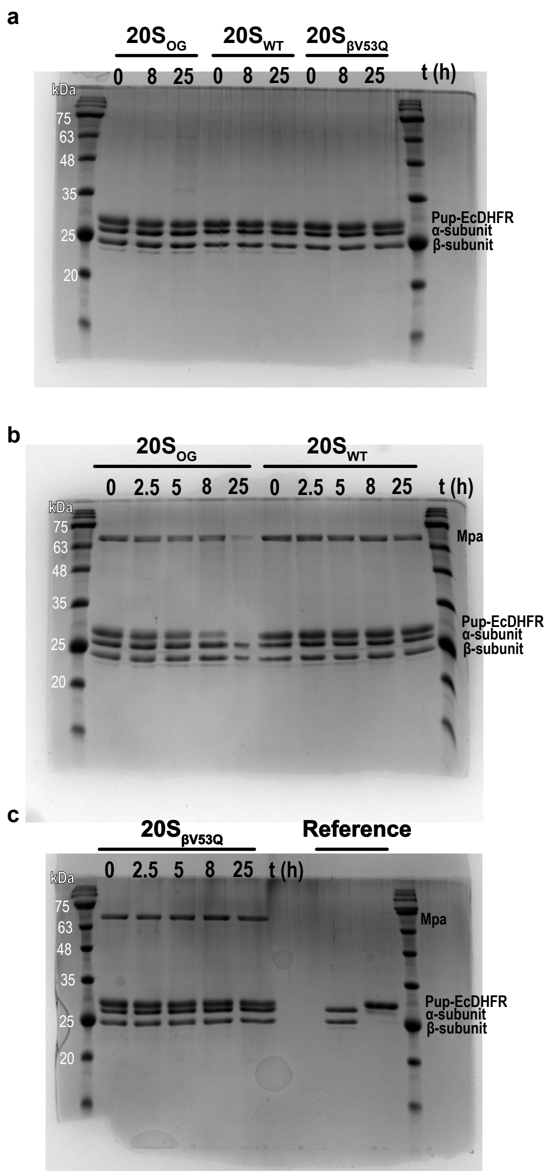

Supplementary Figure 20. The 20S$_{OG}$:Mpa complex is capable of degrading a pupylated, model substrate. SDS-PAGE analysis monitoring the degradation of the model substrate, Pup-EcDHFR, at 37 °C over 25 h reveals that (**a**) substrate degradation is Mpa-dependent; and (**b**, **c**) only the 20S$_{OG}$:Mpa complex is capable of degrading substrate. Timepoints were collected at the indicated hours and reactions were stopped through the addition of Laemmli buffer. Samples were separated on a 20% Tris-glycine gel with Blue Elf ladder (Froggabio) as reference.

Supplementary Table 1. HDX summary

	20S CP WT	20S CP OG	20S CP T1A	20S CP - ixazomib
HDX reaction details	Final D_2O concentration (v/v) = 90%, pH_{corr} 7.4, RT	Final D_2O concentration (v/v) = 90%, pH_{corr} 7.4, RT	Final D_2O concentration (v/v) = 90%, pH_{corr} 7.4, RT	Final D_2O concentration (v/v) = 90%, pH_{corr} 7.4, RT<br>10 μM ixazomib (K_D = 1.0 μM); 1% DMSO
HDX time course (min)	0.167, 1, 5, 15, 60, 180, 1440			
Undeuterated controls	3			
Back-exchange	α-subunit average back-exchange: 32.8% (range: 13.8 – 52.3 %)<br>β-subunit average back-exchange: 33.3% (range: 16.9 – 53.6 %)			
Number of peptides	α-subunit: 78 peptides<br>β-subunit: 60 peptides			α-subunit: 73 peptides<br>β-subunit: 58 peptides
Sequence coverage	α-subunit: 95.2%<br>β-subunit: 91.4%	α-subunit: 97.2%<br>β-subunit: 91.4%	α-subunit: 95.2%<br>β-subunit: 91.4%	α-subunit: 94%<br>β-subunit: 86.7%
Average peptide length/redundancy	α-subunit redundancy: 3.55<br>β-subunit redundancy: 3.04	α-subunit redundancy: 3.55<br>β-subunit redundancy: 3.04	α-subunit redundancy: 3.55<br>β-subunit redundancy: 3.04	α-subunit redundancy: 3.23<br>β-subunit redundancy: 3.04
Replicates	3 technical replicates			
Repeatability	0.24 Da			
Significant differences	0.5 Da			

Supplementary Table 2. HDX summary

	**20S CP$_{WT}$**	**20S CP$_{\alpha Q98K}$**	**20S CP$_{\beta A100S}$**	**20S CP$_{\beta A92\text{-}94G}$**
HDX reaction details	Final D_2O concentration (v/v) = 90%, pH$_{corr}$ 7.4, RT			
HDX time course (sec)	10			
Undeuterated controls	1			
Back-exchange	α-subunit average back-exchange: 32.8% (range: 13.8 – 52.3 %)<br>β-subunit average back-exchange: 33.3% (range: 16.9 – 53.6 %)			
Number of peptides	α-subunit: 73 peptides<br>β-subunit: 73 peptides			α-subunit: 73 peptides<br>β-subunit: 71 peptides
Sequence coverage	α-subunit: 98.0%<br>β-subunit: 97.5%			
Average peptide length/redundancy	α-subunit redundancy: 3.76<br>β-subunit redundancy: 4.06			α-subunit redundancy: 3.76<br>β-subunit redundancy: 4.01
Replicates	3 technical replicates			
Repeatability	0.24 Da			
Significant differences	0.5 Da			

References for Supplementary Information

1. Ortega, A., Amorós, D. & García de la Torre, J. Prediction of Hydrodynamic and Other Solution Properties of Rigid Proteins from Atomic- and Residue-Level Models. *Biophys J* **101**, 892–898 (2011).
2. Atkins, P. *Physical Chemistry*. (W. H. Freeman & Co., New York, 2010).
