# Supplementary material for: Structural basis for allosteric modulation of M. tuberculosis proteasome core particle
Source: Nat Commun. 2025 Apr 1;16:3138. doi: 10.1038/s41467-025-58430-0 (PMC11962144; doi:10.1038/s41467-025-58430-0)
Supplement: Supplementary file 3 — Description of Additional Supplementary Files [file 41467_2025_58430_MOESM3_ESM.pdf]

Description of Additional Supplementary Files

File Name: Supplementary Movie 1
Description: 3DVA component 1 for the $20S_{\beta T1A}$ variant depicting the alternating motion of the α-subunits.

File Name: Supplementary Movie 2
Description: 3DVA component 2 for the $20S_{\beta T1A}$ variant depicting the overall bending of the 20S CP.

File Name: Supplementary Movie 3
Description: 3DVA component 3 for the $20S_{\beta T1A}$ variant depicting the increased flexibility of the switch helices.

File Name: Supplementary Movie 4
Description: 3DVA component 3 for the $20S_{WT}$ variant depicting a similar unwinding of switch helix II.

File Name: Supplementary Movie 5
Description: 3DVA component 3 for the $20S_{OG}$ variant depicting a similar unwinding of switch helix II.

File Name: Supplementary Movie 6
Description: 3DVA component 3 for the ixazomib-bound $20S_{WT}$ variant depicting enhanced stability of the switch helices.

File Name: Supplementary Data 1
Description: Cryo-EM data collection and model building. Attached as an Excel Spreadsheet.

File Name: Supplementary Data 2

Description: Cryo-EM data processing parameters for $20S_{\beta T1A}$ intermediate reconstructions. Attached as an Excel Spreadsheet.
